# Management of an hypopharyngeal fistula following open diverticulectomy for giant Zenker's diverticulum: a case report

**DOI:** 10.3389/fsurg.2025.1714584

**Published:** 2026-01-26

**Authors:** Othmane Bourouail, Abdelilah Hamada, Ali Kada, El Mustapha Halim, Noureddine Njoumi, Mbarek Yaka, Mohammed Elfahssi, Abdelrrahman Elhjouji, Abdelmounaim Aitali

**Affiliations:** Visceral Surgery Service II, Military Hospital of Instruction Mohammed V, Rabat, Morocco

**Keywords:** closure method, esophageal stricture, hypopharyngeal fistula, large Zenker's diverticulum, open drainage

## Abstract

Postoperative hypopharyngeal fistula is an uncommon yet serious complication of open surgery for Zenker's diverticulum, with an incidence of approximately 1%–4%. It is associated with local infection, malnutrition, deterioration of the patient's general condition, and an increased risk of subsequent esophageal stricture. The most valuable therapeutic approach is based on surgical reintervention with reinforced closure, adequate drainage, antibiotic therapy, and optimization of hemodynamic and nutritional parameters. Other methods may also be used, including digestive diversion or conservative management strategies such as radiologically guided drainage. We report the case of a 54-year-old man with a large symptomatic Zenker diverticulum who underwent open mechanical diverticulectomy. The immediate postoperative course was uneventful, and the patient was discharged on day six; however, four days later he was readmitted with painful cervical swelling and signs of infection. Cervical CT with oral contrast demonstrated an extensive hypopharyngeal fistula. Broad-spectrum antibiotic therapy was initiated, and on the second day of hospitalization the patient underwent surgical re-exploration with primary closure of the defect reinforced by a muscular flap and adequate drainage, followed by enteral nutritional support. The postoperative evolution was favorable, with complete closure of the fistula. During follow-up, an esophageal stenosis developed but was successfully managed by endoscopic dilatation. This case highlights the rarity and the management challenges of hypopharyngeal fistula following Zenker's diverticulectomy. It underscores the importance of early recognition and timely surgical intervention using a reinforced closure technique, which is considered the most reliable approach according to current literature for managing fistulas in the hypopharyngeal region. Coordinated postoperative care with rigorous monitoring remains essential to achieve favorable outcomes, despite the potential for long-term sequelae such as esophageal stricture.

## Introduction

Zenker's diverticulum (ZD) is a distinctive esophageal disorder characterized by herniation of the hypopharyngeal mucosa and submucosa through Killian's dehiscence, a muscular weakness located between the cricopharyngeus and thyropharyngeus muscles (1). This outpouching results from increased intraluminal pressure caused by cricopharyngeal muscle hypertonia or reduced compliance, leading to impaired swallowing physiology and progressive dysphagia (1, 2). Open diverticulectomy remains the standard treatment for symptomatic or complicated Zenker's diverticulum, particularly in patients with large pouches or recurrent aspiration (2). Although it generally provides excellent outcomes, this benign condition is not exempt from complications, which can significantly affect postoperative recovery and lead to substantial morbidity with notable impacts on quality of life (3–5).

Among these, hypopharyngeal fistula (HPF) represents a particularly rare but severe condition, with an incidence reported between 1% and 4% (3–7), resulting from suture dehiscence at the posterior pharyngo-esophageal junction and leading to cervical infection, nutritional compromise, and prolonged hospitalization.

Despite its severity, the literature on HPF remains scarce, and optimal management strategies are not clearly established, creating a significant knowledge gap regarding its mechanisms, risk factors, and prevention. In cases involving giant Zenker diverticula, intraoperative dissection can be extensive and technically demanding at the diverticular neck, where wide mucosal mobilization may weaken the wall and predispose to postoperative leakage, as suggested in our patient. Given this condition, could preventive muscular reinforcement of the suture line during the first surgical procedure help decrease the likelihood of fistula development?

This report contributes to the limited evidence on hypopharyngeal fistula following open diverticulectomy, highlighting the surgical challenges posed by large diverticula, the importance of preventive measures and the appropriate therapeutic strategy including reinforced vascularized muscular closure, adequate drainage, antibiotic therapy, and nutritional support.

## Case description

A 54-year-old male patient presented with mild dysphagia, regurgitation, and halitosis persisting for six months, progressively worsening over the past two months, and accompanied by weight loss. His medical history included benign prostatic hyperplasia; there was no relevant family history. Physical examination was unremarkable, and his BMI was 20.2. Laboratory investigations were within normal limits: hemoglobin 13.7 g/dL, leukocyte count 3,800 /mm^3^, and albumin 45 g/L.

**Figure 1:**
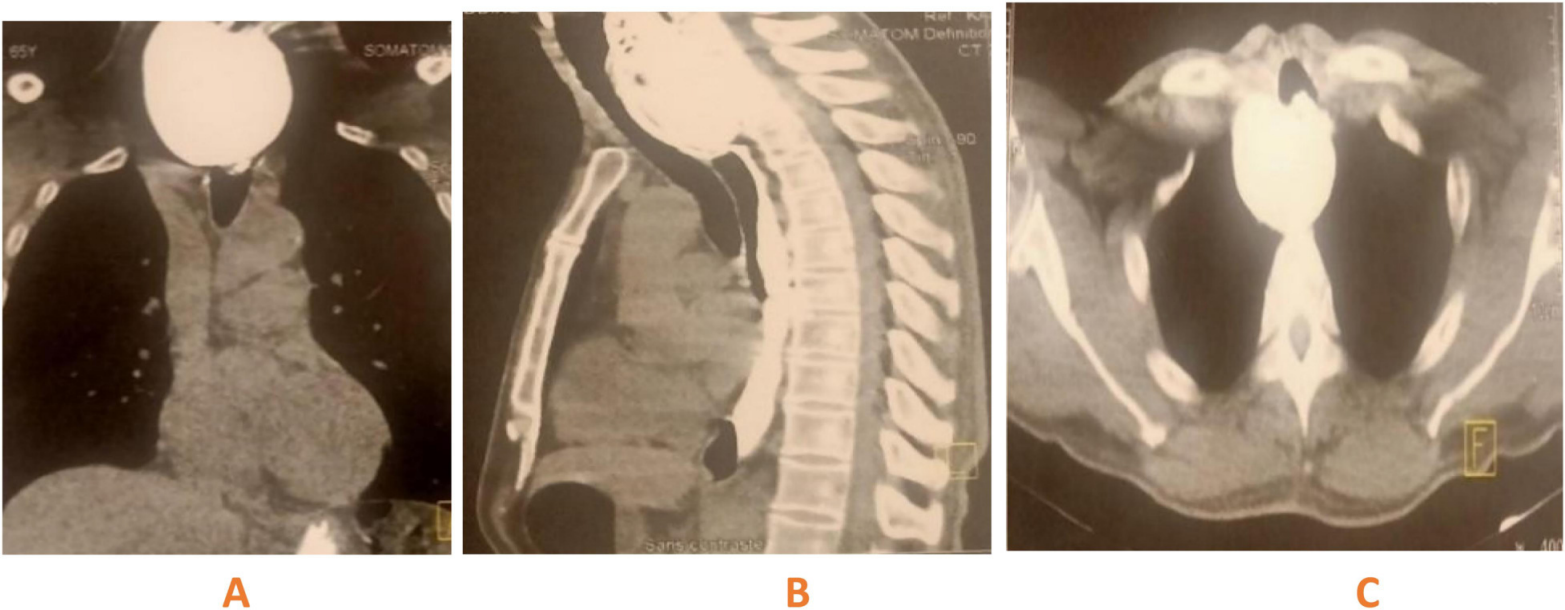
Cervicothoracic CT imaging after oral contrast (**A**: coronal, **B**: sagittal, **C**: axial) reveals a sizable retropharyngeal air-fluid collection measuring 7.2 × 3 cm, corresponding to a diverticular pouch situated anterior to Killian's triangle. The pouch causes anterior displacement of the neck's visceral axis and posterior compression of the esophageal wall.

Upper endoscopy revealed a large diverticular pouch at the upper esophageal opening with food stasis. Given the severity and persistence of symptoms, and to exclude complications, a contrast-enhanced cervical-thoracic CT scan with oral contrast was performed (Figure 1). The imaging revealed a large retropharyngeal air-fluid collection consistent with a diverticular pouch located anterior to Killian's triangle. A calcification measuring 7.2 × 2.7 cm was noted, displacing the esophageal posterior wall. The diagnosis of Zenker's diverticulum (ZD) stage III (Negus classification) was established.

**Figure 2:**
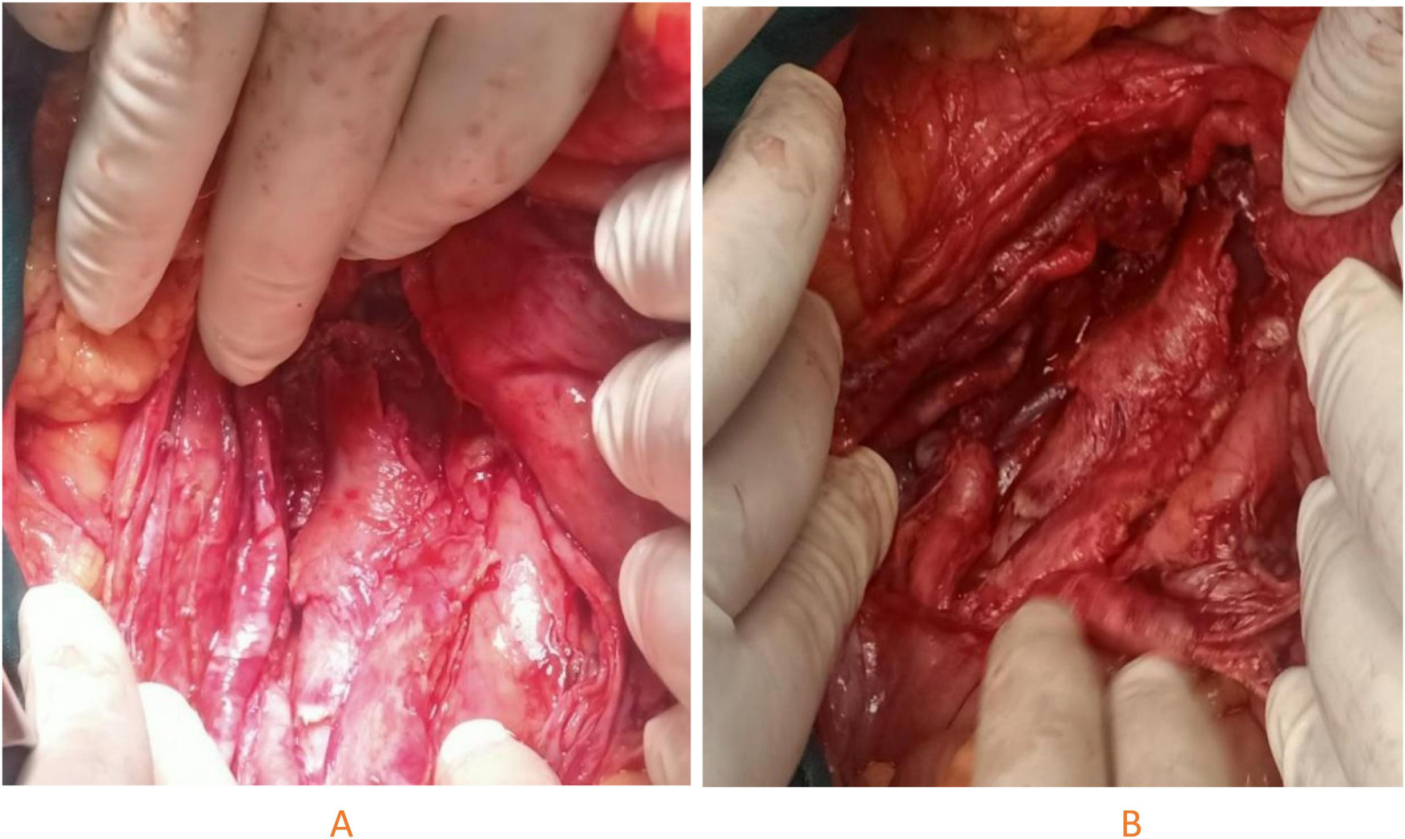
Perioperative images (**A** and **B**) show, after careful and meticulous dissection of the entire pouch up to the neck, the giant zenker's diverticulum adherent to the posterior wall of the pharyngoesophageal junction and extending downward, with displacement of the esophagus inferiorly.

The patient underwent open transcervical diverticulectomy with cricopharyngeal myotomy. He was placed in a supine position with neck hyperflexion and slight right rotation. A 6 cm incision was made along the anterior border of the left sternocleidomastoid muscle. After platysma dissection and division of the omohyoid and strap muscles, the lateral aspect of the thyroid lobe was exposed. The inferior thyroid artery was divided, and dissection continued to the prevertebral fascia. A large, adherent diverticulum compressing the visceral neck axis anteriorly was identified (Figure 2). Given the chronicity of the diverticulum, the sac was densely adherent to the posterior esophageal wall, and the main challenge was dissecting the diverticular neck and adequately exposing the mucosa. The fibrotic tissue surrounding the sac was carefully and meticulously dissected from the posterior hypopharyngeal wall, allowing wide exposure of the mucosa. After identifying the cricopharyngeal muscle, a myotomy was performed by dividing its fibers, together with approximately 1 cm of the upper esophageal circular fibers and 1 cm of the inferior pharyngeal constrictor, using Metzenbaum scissors. The mucosa overlying the sac was then resected, and the diverticulum was divided at its neck with a 30 mm linear stapler (blue reload). The staple line was reinforced with a continuous, slowly absorbable 5-0 PDS suture. A Jost-Redon suction drain was placed anterior to the suture line, and no nasogastric tube was inserted. Intraoperatively, the patient received antibiotic prophylaxis with 1 g of amoxicillin and clavulanic acid. Extubation was uneventful, and no immediate complications were observed.

Postoperative recovery was initially uneventful. The patient continued intravenous antibiotic therapy (3 g/day) and rehydration during hospitalization. Drain output remained serosanguinous with no evidence of air leakage. Oral intake was initiated on postoperative day 3 after both a methylene blue test and a contrast swallow study confirmed free passage without extravasation. The patient gradually resumed a regular diet and was discharged on postoperative day 6 with oral antibiotics continued for seven days and a recommendation for a high-calorie, semi-liquid diet for one week.

**Figure 3:**
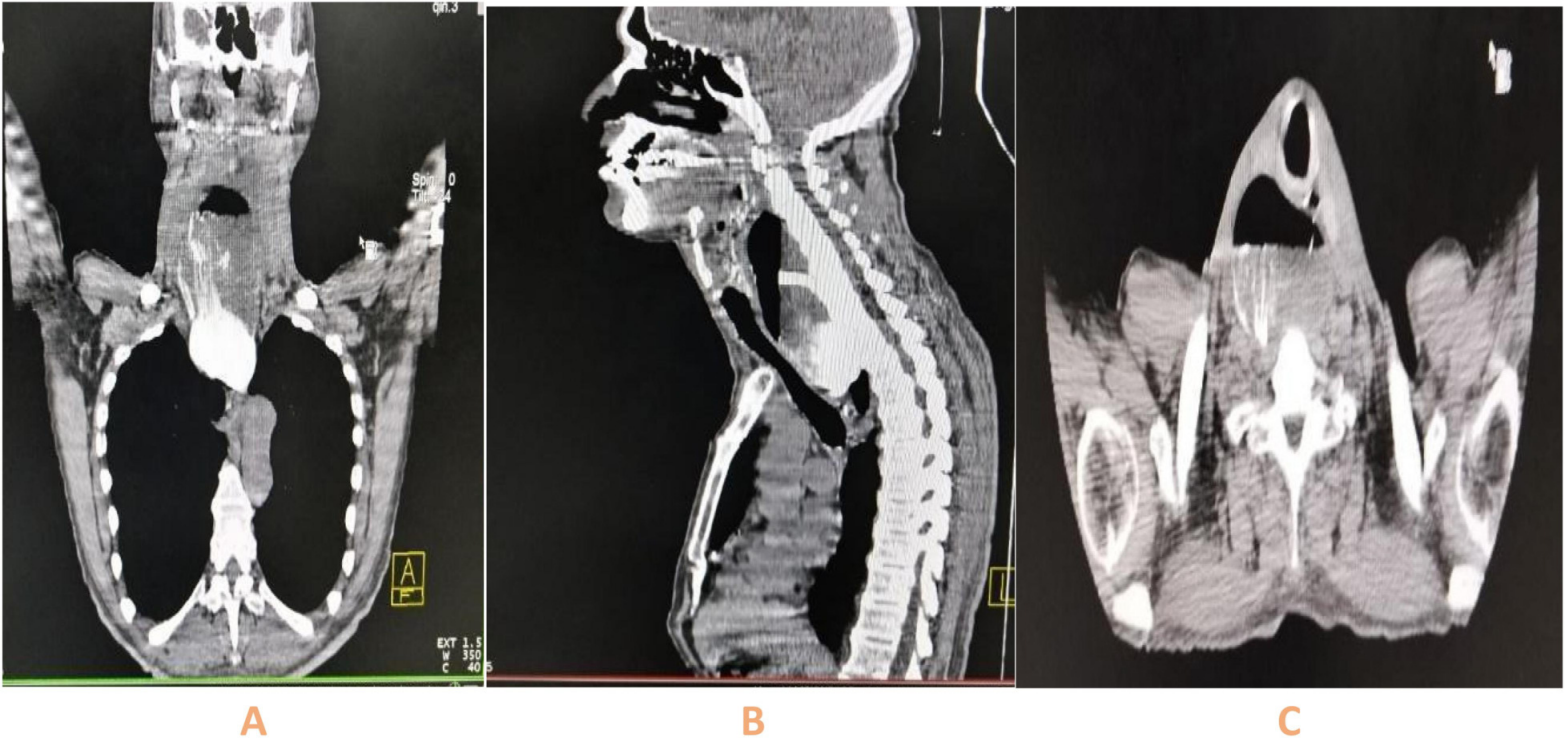
Coronal **(A)**, sagittal **(B)**, and axial **(C)** CT scans with Gastrografin contrast performed 10 days after open diverticulectomy demonstrate a large air-fluid collection adjacent to Killian's dehiscence, measuring 14 × 7.2 × 5.5 cm, lateralized to the right of the esophagus and extending inferiorly to the upper sternum. Extravasation of contrast from the hypopharynx into the collection confirms communication and establishes the diagnosis of a hypopharyngeal fistula.

Four days later, the patient was readmitted with painful right laterocervical swelling. A cervicothoracic CT scan with oral Gastrografin contrast revealed a retropharyngeal air–fluid collection measuring 14 × 7.2 × 5.5 cm, originating from a fistulous tract at Killian's triangle. Oral contrast confirmed leakage from the hypopharyngeal staple line into the collection, consistent with a hypopharyngeal fistula (HPF) (Figure 3). Laboratory findings showed a hemoglobin level of 12.5 g/dL, white blood cell count of 22,000 /mm^3^, C-reactive protein (CRP) of 220 mg/L, and serum albumin of 32 g/L, Serum sodium was 129 mmol/L, potassium 3.1 mmol/L, urea 0.12 g/L, and creatinine 7 mg/L.

The patient was hospitalized with cessation of oral intake, intravenous rehydration, electrolyte supplementation consisting of 1 g of potassium chloride (KCl) and 2 g of sodium chloride (NaCl) and antibiotic therapy consisting of a third-generation cephalosporin, metronidazole, and gentamicin. A decision was made to perform surgical reintervention on the second day of hospitalization. The previous cervicotomy was reopened, revealing an abscess cavity originating from a fistula at the staple line with surrounding necrotic tissue. The cavity was drained, and the fistulous opening was closed using slow-absorbing 4-0 PDS sutures, reinforced with a sternocleidomastoid (SCM) muscle flap. Extensive saline irrigation was carried out, and a large-caliber suction drain was placed. Because the collection extended into the upper mediastinum, a chest tube was inserted for mediastinal drainage.

Antibiotic therapy was continued for a total of 21 days, with a switch to oral administration on day 6. Intravenous rehydration with saline and glucose solutions was maintained even after the resumption of oral feeding. The patient also received Oliclinomel and albumin supplementation to promote healing. Oral feeding was cautiously reintroduced on postoperative day 6, starting with liquids and progressing to a high-calorie, high-protein semi-liquid diet for 14 days. A follow-up cervicothoracic CT scan with oral contrast was performed on days 6 and 10. The scans were normal, showing a hypodense infiltration in the posterior hypopharyngeal region, with the drain in place and no detectable fistula. The cervical drain initially produced serohematic fluid with a progressively decreasing output and was removed around day 10 following clinical, radiological and biological improvement and resumption of oral intake.

Follow-up was scheduled at 1 week, 2 weeks, and 1 month, then every 3 months for the first year, and every 6 months for the following two years. Monitoring is primarily based on clinical evaluation, esophageal transit studies and endoscopy.

Three months after the second surgery, the patient developed an upper esophageal stenosis, clinically manifested by progressive dysphagia and postprandial vomiting. Upper endoscopy revealed a high esophageal stricture with food stasis. The stenosis was successfully treated with endoscopic dilation. The patient is currently under regular follow-up, with contrast esophageal transit studies scheduled every three months during the first year. Endoscopy is reserved for cases of symptom recurrence.

## Discussion and conclusion

A large and chronic Zenker's diverticulum (ZD) presents significant technical challenges, particularly due to the potential for dense adhesions to adjacent cervical structures, which may increase the risk of postoperative complications such as hypopharyngeal fistula. For substantial diverticula, open diverticulectomy with mechanical suture reinforcement combined with cricopharyngeal myotomy remains the standard therapeutic approach, as supported by contemporary evidence (2, 6, 8).

HPF is an infrequent postoperative adverse event, occurring in fewer than 4% of cases after open surgical management and in approximately 1% of cases following endoscopic intervention (1, 4, 6, 9). Its incidence has declined markedly with the introduction of endoscopic techniques and, in particular, mechanically stapled diverticulotomy (9). A recent large-scale analysis of 1,044 patients with Zenker's diverticulum reported an overall hypopharyngeal fistula rate of 0.48% across all operative modalities, representing 7.5% of the documented postoperative complications (35).

Typically, a fistula in this region reflects a disruption of the pharyngoesophageal junction (PEJ) and most commonly manifests clinically as cervical swelling or a palpable cervical mass, indicating local inflammatory involvement (10). Patients often report odynophagia and persistent or progressive dysphagia, further suggesting impaired pharyngeal integrity (11). A pathognomonic sign is the drainage of saliva or ingested material through the cervicotomy wound, confirming an abnormal communication between the pharyngeal lumen and the cervical soft tissues. This external leakage promotes localized infection and inflammation, which may progress to systemic manifestations such as fever and malaise (11). Diagnostic evaluation combines upper gastrointestinal endoscopy to identify suture-line dehiscence with contrast-enhanced radiography or computed tomography to delineate the fistulous tract and assess associated complications (4, 12).

The pathogenesis of postoperative hypopharyngeal fistula (HPF) is multifactorial and influenced by systemic, anatomical, surgical, and postoperative factors. Systemic comorbidities—including diabetes mellitus, advanced age, and obesity—can impair tissue perfusion, disrupt collagen synthesis, and weaken immune responses, thereby increasing susceptibility to suture-line dehiscence (13). Anatomical predispositions, particularly the inherent weakness of Killian's triangle, are further magnified in patients with large Zenker's diverticula, substantially elevating the risk of fistula formation (14). Nutritional status is also a critical determinant; both preoperative and postoperative malnutrition adversely affect tissue repair and promote fistulization. Additionally, postoperative non-adherence to dietary recommendations is a well-recognized contributor to fistula recurrence (13). Surgical factors play an equally pivotal role, as the selection of appropriate stapling devices and the execution of a complete cricopharyngeal myotomy are essential to minimizing the incidence of postoperative HPF (3, 15).

Given the scarcity of published data on the management of hypopharyngeal fistula (HPF) following open diverticulectomy, only a few comparable cases was identified. Weidemann has been described a report of persistent and recurrent hypopharyngocutaneous fistula developed after open diverticulectomy performed in the context of an aberrant right subclavian artery (arteria lusoria), which precluded an endoscopic approach. Multiple attempts at primary fistula closure were unsuccessful. Definitive resolution was obtained only after completion of an initially inadequate cricopharyngeal myotomy, complemented by botulinum toxin injection into the pharyngeal musculature and reinforcement using a platysma muscle flap, resulting in stable closure (3). In contrast, in this present case, a single reoperation consisting of direct fistula closure reinforced by a well-vascularized sternocleidomastoid muscle flap with adequate drainage achieved complete and sustained healing. This comparison highlights the critical importance of employing a robust, vascularized muscle flap during reintervention to strengthen the anatomically vulnerable region and prevent recurrence. Furthermore, proper execution of a complete cricopharyngeal myotomy during the initial diverticulectomy is essential for reducing upper esophageal sphincter pressure and thereby minimizing the risk of postoperative pharyngocutaneous fistula formation or persistence.

Another case reported by Krzysztof Kowalik involved a 44-year-old woman underwent open diverticulectomy for a Zenker's diverticulum and developed a left-sided esophagogastric fistula on postoperative day 5, confirmed by contrast imaging, gastroscopy, and MRI. She required reoperation with fistula excision, drainage of a posterior mediastinal abscess, and multilayer closure of a 1 cm esophageal perforation reinforced by a sternocleidomastoid muscle flap. Eight days later, a free forearm flap reconstruction was attempted, but necrosis and infection led to repeated mediastinal drainage, flap thrombosis with 75% necrosis, and discovery of a new 1.5-cm perforation requiring gastrostomy. The course was further complicated by pneumonia, persistent pneumothorax, and an extensive recurrent fistula (12 × 3.5 cm). After renewed drainage and broad-spectrum antibiotics, gradual improvement was achieved. By day 19, she was stabilized and extubated. She resumed liquid oral intake supplemented by gastrostomy feeding. After six months of hospitalization, she was discharged in stable condition under conservative management and gastroenterology follow-up (10). This case illustrates that, despite being a benign condition, this pathology is not free from complications. Postoperative fistula—whether iatrogenic, due to technical issues, or other contributing factors—can be particularly challenging to manage, especially when associated with infection extending into the mediastinum. Its management therefore requires a rigorous and patient-tailored therapeutic strategy, combining conservative measures (drainage, antibiotics, intensive care) and, when necessary, repeated surgical interventions or even diversion procedures.

As part of a critical analysis of the possible causes of the fistula, several factors were suspected to have contributed to its occuring in our patient. First, mucosal fragility at the level of the pharyngeal collar, related to a wide and difficult dissection, may have played a role. This difficulty was due to the large size of the diverticulum, which displaced the visceral axis and adhered strongly to the posterior wall of the esophagus, particularly if the transection and suturing were performed on an area of weakened mucosa. We should also consider the possibility of unrecognized peridiverticular tissue trauma, a localized hematoma, or excessive tension on the suture line (16). Additionally, the TA 45 mm stapler with blue reload, used twice, may not have ensured optimal closure, potentially favoring dehiscence despite reinforcement suturing. According to some authors, one of the disadvantages of the stapling technique is the incomplete division of the diverticular septum, resulting in a residual sac due to the tip of the stapler, which is not reachable by the blade (15). Finally, although the patient reported having followed postoperative instructions, the early intake of a fatty and solid meal may have exerted excessive pressure on the suture, contributing to its opening. It should be noted that the patient had initially been placed on a strict semi-liquid, high-calorie diet for one week and was scheduled for follow-up consultation to allow a gradual progression to solid food.

Our case underscores the high risk associated with open surgery in the setting of a large Zenker's diverticulum and the multifactorial pathogenesis of fistula formation. Such procedures demand a highly experienced surgical team and a hospital environment equipped with appropriate technical and perioperative support to ensure safe management and optimal outcomes. Strict adherence to established surgical protocols is crucial, including comprehensive preoperative assessment of comorbidities and careful evaluation of the patient's nutritional status. This assessment should ensure a normal body mass index and adequate serum albumin levels, with preoperative nutritional support provided if deficiencies are identified. Systematic antibiotic prophylaxis is also recommended to minimize the risk of perioperative infection. During the procedure, meticulous dissection of the diverticular sac is essential, particularly in large or chronic diverticula where dense adhesions to the posterolateral cervical visceral axis are frequently encountered. Following sac excision, thorough evaluation of the residual mucosa is critical to ensure its viability and completeness. Mechanical closure with a linear stapler must be executed with high precision to achieve full mucosal division and a secure staple line. A complete cricopharyngeal myotomy is mandatory to decrease intraluminal pressure and thereby reduce mechanical stress on the staple line, minimizing the risk of postoperative fistula. When indicated, prophylactic reinforcement with a well-vascularized muscle flap should be incorporated during the initial operation to strengthen the repair. Adequate postoperative drainage further contributes to the prevention of fistulous complications, and all surgical instruments must be fully functional, preferably new, and maintained in strict sterile conditions to optimize surgical safety (17). Postoperatively, close and continuous monitoring is essential, including vital signs (temperature, blood pressure, pulse, oxygen saturation), optimization of hemodynamics, particularly in patients with cardiovascular or respiratory comorbidities, and correction of hydro-electrolytic disturbances. Perioperative nutritional support, including parenteral nutrition and albumin supplementation, should be provided in cases of documented deficits. Oral intake should remain withheld until radiological and biological confirmation of suture integrity. Dietary progression and management of drainage must be gradual and strictly supervised by the treating surgeon prior to drain removal and patient discharge. Finally, the patient should be informed of the potential risk of anastomotic fistula and instructed to promptly contact the surgical team if any warning signs occur.

Currently, no consensus or standardized protocol exists in the literature regarding the optimal timing or specific surgical techniques for the management of hypopharyngeal fistulas, whether of benign or malignant etiology. Most authors therefore rely on analyses of their own clinical experience to propose individualized or algorithmic management strategies. However, Zenker's diverticulectomy is exceedingly rare, and postoperative fistulas in this context are infrequently reported, limiting the ability to establish a well-defined therapeutic approach—particularly given that most studies address the incidence of postoperative fistulas rather than their management. This underscores the necessity of documenting such cases to contribute to the available literature and facilitate future evidence-based analyses (10, 18). In cases of hypopharyngeal fistula (HPF), revision surgery with direct fistula closure is considered the primary surgical approach, as recommended in the literature (13). Management requires thorough open drainage and reinforced closure of the defect, including debridement of devitalized and infected tissue, secure closure of the fistulous tract, and reinforcement with a well-vascularized tissue flap (19–21). In certain instances, the infectious process may extend into the superior mediastinum, creating a risk of life-threatening airway or vascular compromise; therefore, adequate mediastinal drainage, often with the placement of a thoracic tube, is indicated (21). In cases with extensive infection involving intrathoracic or intra-abdominal structures, enteral diversion techniques such as gastrostomy or jejunostomy may be employed to limit contamination, maintain nutritional support, and facilitate fistula healing (11, 22).

In reinforced closure procedures, commonly utilized muscle flaps include the sternocleidomastoid (SCM), sternohyoid, and sternothyroid muscles (17–21). A study by Naghibzadeh, conducted between March 1989 and February 2005 on 88 patients with hypopharyngeal fistulas following pharyngectomy for malignant tumors, evaluated the efficacy of muscle flap reinforcement in preventing fistula recurrence. Among these patients, 37 underwent closure with a muscle flap, whereas 41 were managed without a flap. Fistula recurrence occurred significantly more frequently in the non-flap group (8 cases, 15.7%) compared to the flap group (1 case, 2.7%), with the difference reaching statistical significance (*p* < 0.001) (23). Similarly, a retrospective study by Ting-Han Chiu, including patients with hypopharyngeal cancer who developed pharyngocutaneous fistulas between 2017 and 2021, reported that the majority of cases—acute (1 case), subacute (4 cases), and chronic (5 cases), representing 58.8%—required muscle flap reconstruction using various techniques, with favorable outcomes (18). These findings underscore the critical role of muscle flap reinforcement as an effective therapeutic strategy in the management of hypopharyngeal fistulas, regardless of whether the etiology is benign or malignant.

However, the reinforced closure technique also warrants critical consideration regarding its indications and the underlying pathology. Hypopharyngeal fistulas arising in benign conditions differ from those occurring in oncologic settings, where patients frequently present with malnutrition, impaired general condition, and increased surgical complexity. In benign cases, a conservative approach can be considered appropriate, provided that patients are carefully selected, closely monitored, and that timely surgical intervention is instituted if conservative measures fail. This therapeutic approach may be valid in patients with small, early-detected fistulae without significant necrosis or infection (11, 16, 24), when diagnosis is delayed and extensive inflammation obscures surgical planes, drainage alone may be preferable initially (10, 11, 24). Non-operative treatment, including a strict fasting period of 72 h followed by gradual reintroduction of a liquid diet, broad-spectrum antibiotics for 7–14 days, and parenteral nutrition, has demonstrated efficacy in select cases with mild symptoms and no systemic infection (11, 16, 25). However, vigilant postoperative surveillance is mandatory, as complications such as upper esophageal stenosis may develop even after fistula resolution.

Several studies have identified risk factors predisposing to the development of esophageal strictures, including cardiovascular disease, significant intraoperative blood loss, the type and location of the anastomosis (hand-sewn vs. stapled, cervical vs. thoracic), as well as postoperative complications such as leakage, fistula formation, or infection at the anastomotic site (26, 27). The gastrointestinal mucosa functions as a critical epithelial barrier protecting the digestive tract. When an esophageal mucosal defect occurs, the underlying submucosal tissues are directly exposed to irritants such as food, saliva, gastric acid, and various bacteria and fungi, resulting in persistent tissue injury. Chronic inflammatory responses, coupled with myofibroblast proliferation and fibrosis, have been shown to decrease esophageal luminal compliance and elasticity, thereby promoting stricture formation (28–30).

Additional factors may contribute to stricture development, including tension at the suture line, which can cause ischemia or mucosal edema. Esophagitis secondary to food stasis may further exacerbate luminal narrowing. A dysphagia score developed by Dakkak et al. in a study of 64 patients demonstrated that stricture diameter accounted for only 30% of the dysphagia score, whereas esophagitis and other factors contributed to the remaining 70% (31). In our case, stricture formation was likely facilitated by a hypopharyngeal fistula, infection, inflammatory processes, probable ischemic injury to the esophageal mucosa, and the development of scar tissue.

Several therapeutic strategies have been described for the management of esophageal strictures. For benign strictures, first-line treatment consists of endoscopic mechanical dilation, including balloon dilation, Savary bougies, Eder-Puestow olive dilators, or fluoroscopic balloon dilation (26, 27, 32). The majority of patients achieve improvement after up to five dilation sessions; however, approximately 10% require repeated dilations to maintain a stable esophageal lumen (33). In refractory strictures, adjunctive options may include intralesional corticosteroid injections combined with dilation, esophageal stenting, or endoscopic stricturoplasty techniques, with variable success rates reported (27, 33). When these approaches fail, self-dilation using Maloney dilators represents a safe and potentially effective alternative (34). Finally, as emphasized by Van Boeckel and Siersema, surgical intervention should be reserved as a last-line option (34).

Open surgery for a giant Zenker's diverticulum is a complex and high-risk procedure that requires an experienced surgical team, appropriate technical facilities, and an adequately equipped hospital setting. A thorough perioperative evaluation, including identification of comorbidities and assessment of nutritional status, is essential, along with the implementation of nutritional support, hemodynamic optimization, and systematic antibiotic prophylaxis when indicated. HPF following surgery for a large Zenker's diverticulum is rare, typically presenting with hypopharyngeal infection, cervical swelling, and leakage of saliva or food. In managing this complication, prevention is paramount, the complete myotomy and the use of a muscular flap should be considered when dissection is difficult, when the risk of tissue injury is high, or when the mucosa appears compromised (17). Reinforced closure using a vascularized muscle flap, combined with effective drainage, antibiotic therapy and nutritional support if it is needed, remains the safest and most effective approach reported to date. However, there is currently no consensus or standardized protocol for the treatment of hypopharyngeal fistulas, whether arising in benign or malignant conditions, underscoring the value of case reports in building an evidence base and guiding future recommendations. These fistulas may lead to hypopharyngo-esophageal stenosis, which can be recurrent and difficult to manage, often requiring multiple endoscopic dilatations or, in some cases, surgical intervention.

This report has several inherent limitations that must be acknowledged. First, it is based on a single-case observation, which restricts the generalizability of our findings. While the case provides valuable insights into the management of hypopharyngeal fistula following open diverticulectomy, conclusions drawn from a solitary instance cannot be assumed to apply broadly to all patients with similar complications. Second, the follow-up period in this study was relatively limited. Longer-term outcomes, including the risk of recurrence, late complications, or functional sequelae such as dysphagia, remain unknown. Extended monitoring would be necessary to fully assess the durability and safety of the management approach used. Finally, our report lacks a direct comparison with alternative treatment techniques, such as endoscopic management, conservative therapy, or different surgical closure methods. Without comparative data, it is difficult to determine whether the approach described offers superior efficacy, fewer complications, or better functional outcomes relative to other strategies. Despite these limitations, this case contributes valuable clinical insights and highlights important considerations for the recognition and management of hypopharyngeal fistulas after open diverticulectomy.

## Data Availability

The original contributions presented in the study are included in the article/Supplementary Material, further inquiries can be directed to the corresponding author.
